# TLR7 Signaling Drives the Development of Sjögren’s Syndrome

**DOI:** 10.3389/fimmu.2021.676010

**Published:** 2021-05-24

**Authors:** Yawen Wang, Annie Roussel-Queval, Lionel Chasson, Noël Hanna Kazazian, Laetitia Marcadet, Andrianos Nezos, Michael H. Sieweke, Clio Mavragani, Lena Alexopoulou

**Affiliations:** ^1^ Aix Marseille Univ, CNRS, INSERM, CIML, Marseille, France; ^2^ Departments of Physiology and Pathophysiology, National and Kapodistrian University of Athens School of Medicine, Athens, Greece; ^3^ Center for Regenerative Therapies Dresden (CRTD), Technische Universität Dresden, Dresden, Germany; ^4^ Max-Delbrück-Centrum für Molekulare Medizin in der Helmholtzgemeinschaft (MDC), Berlin, Germany

**Keywords:** Sjögren’s syndrome (SS), TLR7, innate immunity, autoimmunity, TLR8-deficient mice, dendritic cells, transgenic mice

## Abstract

Sjögren’s syndrome (SS) is a chronic systemic autoimmune disease that affects predominately salivary and lacrimal glands. SS can occur alone or in combination with another autoimmune disease like systemic lupus erythematosus (SLE). Here we report that TLR7 signaling drives the development of SS since TLR8-deficient (TLR8ko) mice that develop lupus due to increased TLR7 signaling by dendritic cells, also develop an age-dependent secondary pathology similar to associated SS. The SS phenotype in TLR8ko mice is manifested by sialadenitis, increased anti-SSA and anti-SSB autoantibody production, immune complex deposition and increased cytokine production in salivary glands, as well as lung inflammation. Moreover, ectopic lymphoid structures characterized by B/T aggregates, formation of high endothelial venules and the presence of dendritic cells are formed in the salivary glands of TLR8ko mice. Interestingly, all these phenotypes are abrogated in double TLR7/8-deficient mice, suggesting that the SS phenotype in TLR8-deficient mice is TLR7-dependent. In addition, evaluation of TLR7 and inflammatory markers in the salivary glands of primary SS patients revealed significantly increased *TLR7* expression levels compared to healthy individuals, that were positively correlated to *TNF*, *LT-α*, *CXCL13* and *CXCR5* expression. These findings establish an important role of TLR7 signaling for local and systemic SS disease manifestations, and inhibition of such will likely have therapeutic value.

## Introduction

Sjögren’s syndrome (SS) is a chronic systemic autoimmune disease characterized by infiltration and destruction of exocrine glands, predominantly the salivary and lacrimal glands, that leads to xerostomia (dry mouth) and keratoconjunctivitis sicca (dry eyes) (1). Apart from glandular symptoms, fatigue and arthralgia are also common, and systemic complications involving the respiratory, vascular, neurological and nervous systems, affects one third of patients (2). The prevalence of the disease is about 0.5% in the general population and it is most commonly seen in middle-aged women, with a considerable sex bias of 9:1 female to male ratio (1). SS can develop as an entity alone, termed “primary” SS or it can occur together with another autoimmune disease, like systemic lupus erythematosus (SLE), rheumatoid arthritis or systemic sclerosis, termed “secondary” or “associated” SS (3). The etiology of SS is multifactorial, where genetic, epigenetic, hormonal and environmental factors are thought to interact (4, 5). Among the environmental factors, microbial infections, particularly by viruses such as Epstein-Barr virus, cytomegalovirus, hepatitis C virus and retroviruses are considered as an important trigger for SS (4).

Little is known about the mechanisms that support SS etiology and the corresponding early and sustained molecular events in SS disease. At the same time, growing evidence points towards a significant role of innate immune detection of nucleic acids by TLRs in the development of autoimmunity (6). Nucleic acids of exogenous (microbial) or endogenous (nuclear or mitochondrial) origin are detected by nucleic acid receptors in the cytoplasm and induce pro-inflammatory cytokines and type I IFN production. Human endosomal TLRs consist of TLR3 that senses double-stranded RNA (7), TLR7 and TLR8 that detect single-stranded RNA and TLR9 that recognizes DNA containing unmethylated CG dinucleotides (8). Interestingly, the corresponding murine TLRs detect similar ligands, with the exception of murine TLR8 that is unable to sense ssRNA due to the lack of five amino acids in its extracellular part (9). Despite the fact that murine TLR8 does not detect ssRNA, we demonstrated previously that it plays an important biological role in restraining TLR7 signaling on dendritic cells, but not on B cells or macrophages (10, 11). Similarly, human TLR8 can also restrain mouse TLR7, as it was reported in transgenic mice that express human TLR8, whereas high expression of human TLR8 led to severe inflammation and premature death, while low expression caused increased susceptibility to collagen-induced arthritis (12). Although the endosomal localization isolate TLR3, 7, 8 and 9 away from self-nucleic acids in the extracellular space, still self-RNA or -DNA can become a potent trigger of cell activation when transported into TLR-containing endosomes and can lead to sterile inflammation and autoimmunity (6). Particularly, endosomal TLR7 has been shown to play a critical role in the development of SLE, while its role in SS remains poorly understood. Indeed, increased TLR7 signaling has been associated with SLE development both in humans and mice, while in various SLE mouse models depletion of TLR7 led to the amelioration or even prevention of lupus disease (10, 11, 13–18). TLR ligation leads to the recruitment of the adaptor molecule MyD88, except of TLR3 that recruits TRIF, and triggers the activation of transcription factors, which induce the release of pro-inflammatory cytokines, type I IFNs and other proinflammatory mediators that contribute to the development of autoimmunity (19). MyD88 is ubiquitously expressed by all immune cells and is required for TLR signaling, except of TLR3. Interestingly, in the NOD.B10sn-H2b/j (NOD.B10) mouse model of primary SS, deletion of the MyD88 signaling pathway leads to attenuated SS disease, suggesting that MyD88 is a crucial mediator of local and systemic SS disease manifestations (20).

Salivary gland dysfunction in SS is attributed to both a systemic and a localized autoimmune response within the salivary gland. The systemic autoimmunity in SS is highlighted by the detection of antibodies, mainly towards ribonucleoproteinic complexes such as anti-SSA (anti-Ro) and anti-SSB (anti-La), which serve as markers for clinical diagnosis (21). The presence of organized ectopic lymphoid structures (ELS) that contain infiltrating T and B lymphocytes, dendritic cells (DCs) and macrophages, within the salivary glands are indicative of a localized immune response in the disease (1). The morphology of these ELS ranges from aggregates of lymphocytes, to well organized tertiary lymphoid organs (TLOs) containing distinct T and B cells zones, DCs, high endothelial venules (HEV), lymphatic vessels and B cell follicles with germinal centers (22). The development of ELS in organs that are specifically targeted by autoimmune disease suggest that they function as local sites of antigen presentation by DCs and areas of lymphocyte activation (22). Several cytokines/chemokines and their corresponding receptors are involved in the formation of TLOs, including lymphotoxins (LTa3 and LTa1b2), TNF, CXCL13, CCL19 and CCL21 (23).

The importance of TLR7 signaling in the development of SLE is well established, however, its role in SS development is poorly understood. Moreover, SLE and SS are two closely related autoimmune diseases and a possible connection between the mechanisms that lead to SLE and SS has been suggested based on some common features of the two diseases, including epidemiological characteristics, clinical manifestations, serological profiles and genetic risk factors (24). Despite the differences in the immune system between humans and mice, murine models constitute an advanced tool for modelling pathogenic mechanisms of complex diseases like SLE or SS (25). In this context we have shown previously that TLR8-deficiency in C57BL/6 mice leads to lupus development due to increased TLR7 expression and signaling by DCs, and that this phenotype can be exacerbated under high fat diet conditions due to the additional increase of TLR7 signaling (10, 11, 26). Here we demonstrate a critical role for TLR7 signaling in the pathogenesis of associated SS in TLR8-deficient mice that is characterized by sialadenitis, increased SSA and SSB autoantibody production, immune complex deposition, increased cytokine production and development of ELS in salivary glands, as well as lung inflammation. Interestingly, in human salivary gland tissue of SS patients we found increased *TLR7* expression that was positively correlated to *TNF*, *LT-α*, *CXCL13* and *CXCR5* expression levels.

## Materials and Methods

### Mice

TLR8ko and double TLR7/8ko mice were generated as described previously (10, 27). Both TLR-deficient mouse lines were backcrossed on the C57BL/6 background for more than 10 generations. In order to normalize the microbiota between the TLR-deficient mice and WT controls and because the TLR7 and TLR8 genes are both located in the X chromosome, female age-matched TLR-deficient mice and their respective WT control mice were derived by mating littermate TLR-heterozygous female mice with WT or TLR-deficient male mice (e.g., TLR8^X+X−^ x TLR8^X+Y^, or TLR8^X−Y^). Mice were allowed to consume water and pellet shew ad libitum. Mice were housed under specific pathogen-free conditions at the Center d’Immunologie de Marseille-Luminy. All animal studies were performed in accordance with institutional guidelines for care and use of laboratory animals and with protocols approved by the Comité National de Réflexion Ethique sur l’Expérimentation Animale.

### Histology and Immunofluorescence

For histopathology studies, salivary gland and lung tissues were collected. SG were fixed in AntigenFix (MMFRance, Francheville, France) for 2h, washed with phosphate buffer, dehydrated in 30% sucrose in PBS for an overnight, embedded in OCT-compound and frozen in liquid nitrogen. Sections were cut on cryostat at 10 μm, thaw-mounted on gelatinized slides and stained with H&E or processed for immunofluorescence staining. Antibodies that were used were: hamster anti-CD11c (clone N418, BD Biosciences) followed by anti-hamster IgG/AF488 (polyclonal, Jackson Immunoresearch), rat anti-CD3 (Biorad) followed by anti-rat IgG/AF594, rat anti-CD45R/AF647 (clone RA3-6B2, BD Biosciences), rat anti-HEV marker/AF488 (clone Meca-79, eBioscience), donkey anti-IgG/AF488 (Invitrogen), and anti-IgM/AF633 (Invitrogen). Lungs were fixed in formalin, embedded in paraffin and stained with H&E. Slides were scanned using Pannoramic Scan (3D Histech) and Image J was used to measure the lymphocytic infiltration present in the tissue. This was quantified by the division of the area of infiltration by the total tissue area examined.

### Serological Analysis

For detection of autoantibodies against SSA, SSB and RNA on serum samples, Nunc plates were precoated with 1.0 µg/ml Ro (SSA), 1.0 µg/ml La (SSB) antigen (Arotec Diagnostics), or 5µg/ml RNA (purified from mouse liver) for an overnight. Plates were incubated for 1h with blocking buffer (PBS, 1% BSA), mouse sera were applied at a dilution 1:100 and the assay was developed using anti-mouse IgG (H+L)-horseradish peroxidase (Southern Biotech).

### RNA Isolation and Q-PCR

Total RNA from murine SMG was isolated with TRizol reagent. RNA was reverse transcribed with Superscript II reverse transcriptase (Invitrogen) and Q-PCR was performed as described previously (10). For human minor SG, RNA extraction, cDNA synthesis and Q-PCR were done as described previously (28). The primers used for Q-PCR are listed in **Supplementary Table 1**.

### Subjects

Minor salivary gland (MSG) biopsies were obtained from 40 SS patients and 8 sicca controls at the Department of Pathophysiology, School of Medicine at the National and Kapodistrian University of Athens, Athens, Greece as a routine part of the diagnostic evaluation for SS. A written informed consent was received from participants prior collection of the samples. None of the SS patients were complicated by lymphoma. The biopsies were immediately frozen at -80°C. Focus score was determined for each MSG biopsy sample, as previously described (29). SS patients and sicca controls were females with mean age ± SD: 59 ± 16 and 53 ± 8 years old, respectively. SS patients were classified according to the 2016 ACR criteria (30). The sicca control group included individuals complaining of sicca symptoms, not fulfilling the SS criteria.

### Statistics

**Figure 1:**
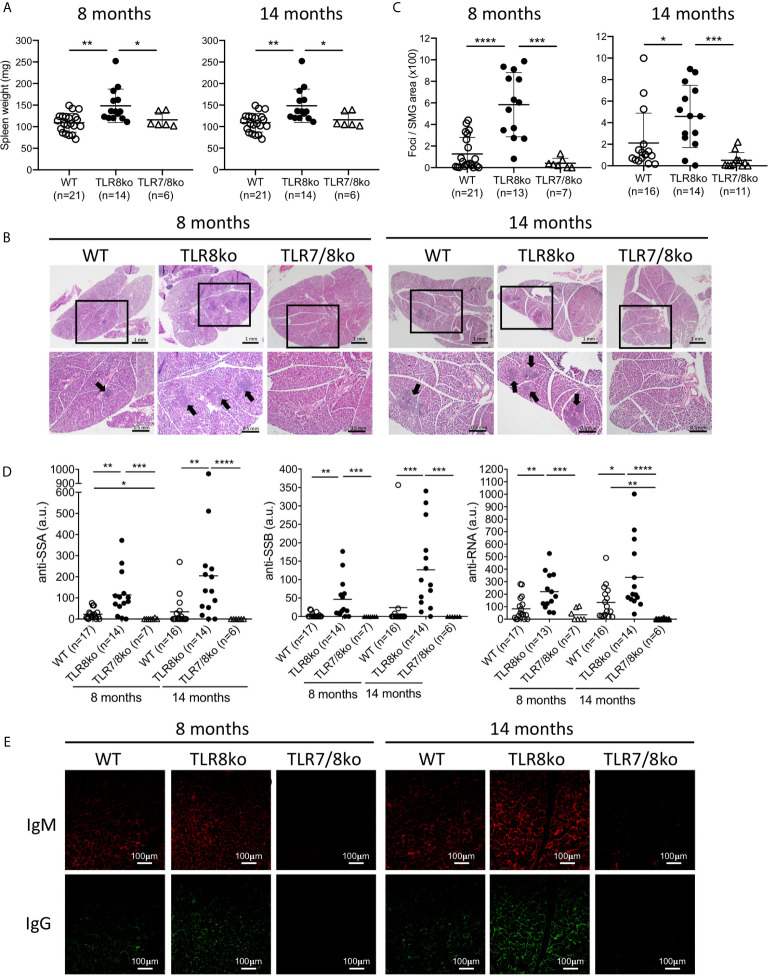
TLR8ko mice develop TLR7-dependent SS. Spleens and salivary gland were harvested from 8- and 14-months-old TLR8ko, TLR7/8ko and WT female mice. **(A)** Spleen weight of TLR8ko, TLR7/8ko and WT mice. **(B)** Salivary gland tissues were sectioned and representative areas of H&E stained sections are shown. Panels in upper row magnification x1, panels in lower row magnification x4 of the black frames indicated in the upper row panels. Black arrows denote lymphocytic infiltration. **(C)** Pathological scores of SG were evaluated and results are expressed as mean +/- SD of total foci area versus SG area x 100. Scale bars: 0.5 mm. **(D)** Levels of anti-SSA, anti-SSB and anti-RNA antibodies in sera of 8- and 14-months-old TLR8ko, TLR7/8ko and WT female mice were evaluated by ELISA. **(E)** Representative microphotographs of immunofluorescence detection of IgG and IgM in SG sections from TLR8ko, TLR7/8ko and WT female mice. Scale bars: 100 μm. Statistical analysis was done by Kruskal-Wallis followed by Wilcoxon rank sum tests and correction for multiple comparisons using the Benjamini-Hochberg method. In **(A, B, D)** each time point represents the value of one mouse and horizontal bars denote mean. In **(A–D)** pooled data from 4 independent experiments, and in **(E)** data are representative of 2 independent experiments with 3-5 mice per genotype. *P < 0.05, **P < 0.01, ***P < 0.001, ****P < 0.0001.

**Figure 2:**
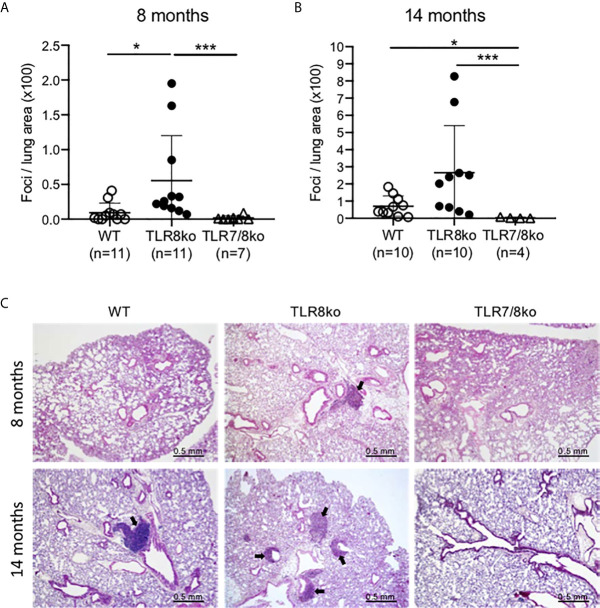
Lung inflammation in TLR8ko mice. Lung inflammation scoring at **(A)** 8-months and **(B)** 14 months old WT, TLR8ko and TLR7/8ko mice. **(C)** Representative micrographs of lungs from 8- and 14-months old WT, TLR8ko and TLR7/8ko mice. H&E-stained lung sections show inflammatory foci (arrows). Scale bars: 0.5 mm. Statistical analysis was done by Kruskal-Wallis followed by Wilcoxon rank sum tests and correction for multiple comparisons using the Benjamini-Hochberg method. Data are representative of 2 independent experiments combined. *P < 0.05, ***P < 0.001.

**Figure 3:**
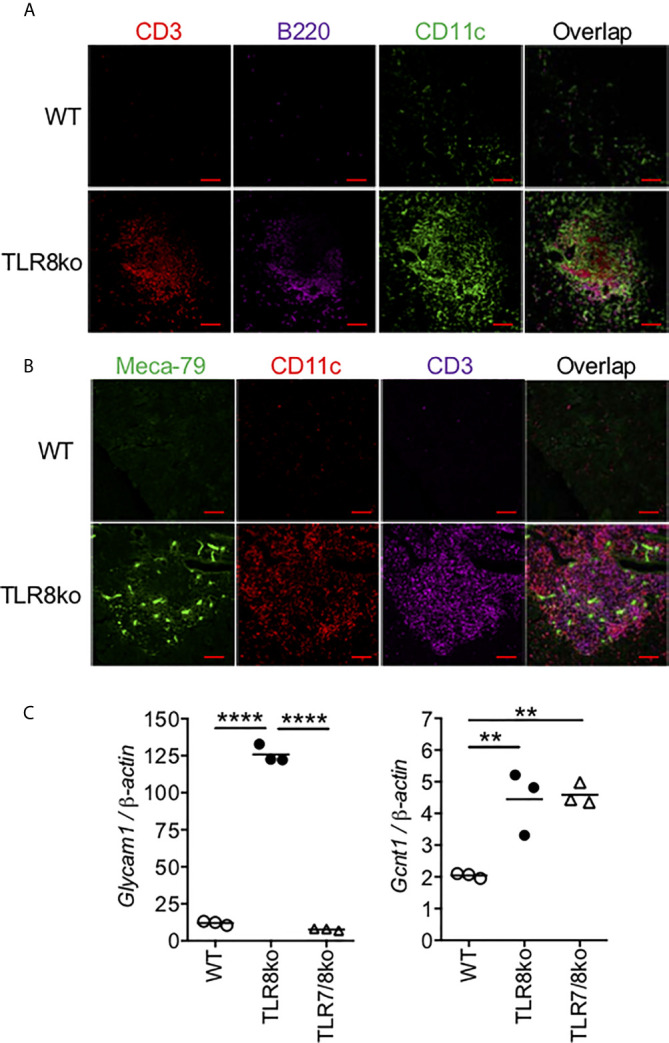
Identification of TLS in the infiltrates of TLR8ko SMG. Immunofluorescent microscopic analysis of lymphocyte infiltrates in SMG of 8-months old TLR8ko mice. Frozen sections were stained with fluorescent labelled **(A)** CD3 for T cells (red), B220 for B cells (violet) and CD11c for DCs (green) and **(B)** Meca79 for HEV (green), CD11c for DCs (red) and CD3 for T cells (violet). Scale bars: 100 μm; magnification x20. **(C)** Expression of the HEV markers *Glycam1* and *Gcnt1* was evaluated in the SMG of WT, TLR8ko and TLR7/8ko mice by Q-PCR. Plots represent mean ± SD of triplicates, with n=5-6 mice combined per genotype. Statistical analysis was done by one-way ANOVA followed by Tukey’s multiple-comparison test. **(A–C)** Results are representative of two independent experiments with 5-6 mice per genotype and per experiment. **P < 0.01, ****P < 0.0001.

**Figure 4:**
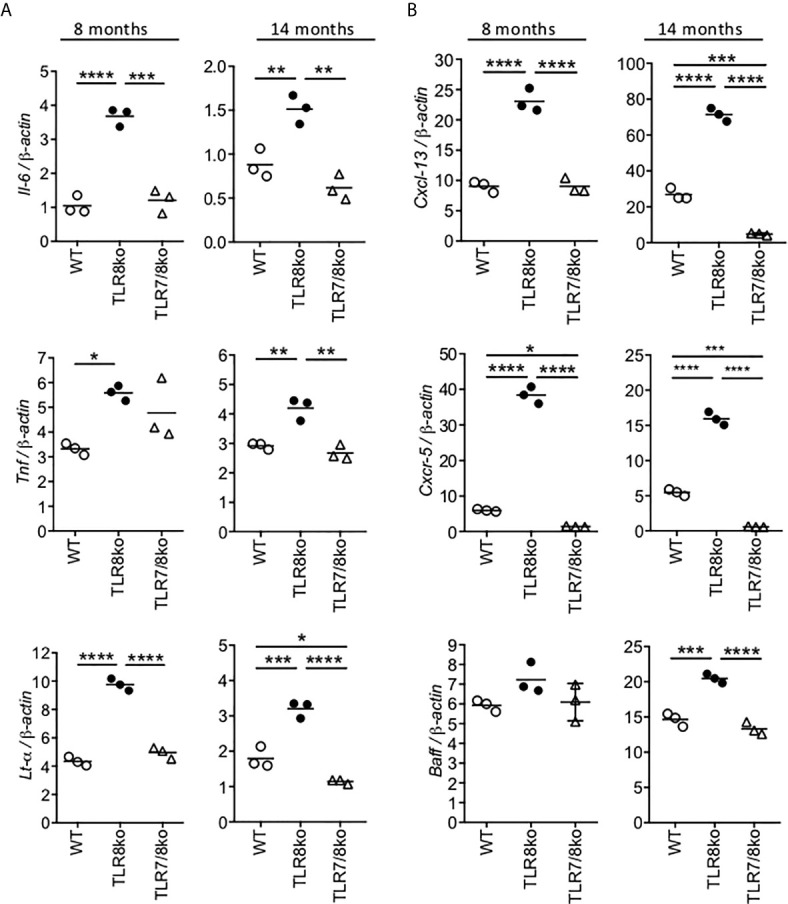
Increased levels of cytokines and B cell markers in TLR8ko SMG. Expression of **(A)** *Il-6*, *Tnf* and *Lt-α* and **(B)** *Cxcl13*, *Cxcr5* and *Baff* mRNA levels were evaluated in the SMG of 8- and 14-months old female WT, TLR8ko and TLR7/8ko mice by Q-PCR. Plots represent mean ± SD of triplicates, with n=5-6 mice combined per genotype. Statistical analysis was done by one-way ANOVA followed by Tukey’s multiple-comparison test. Data are representative of at least two independent experiments. *P <0.05, **P < 0.01, ***P < 0.001, ****P < 0.0001.

**Figure 5:**
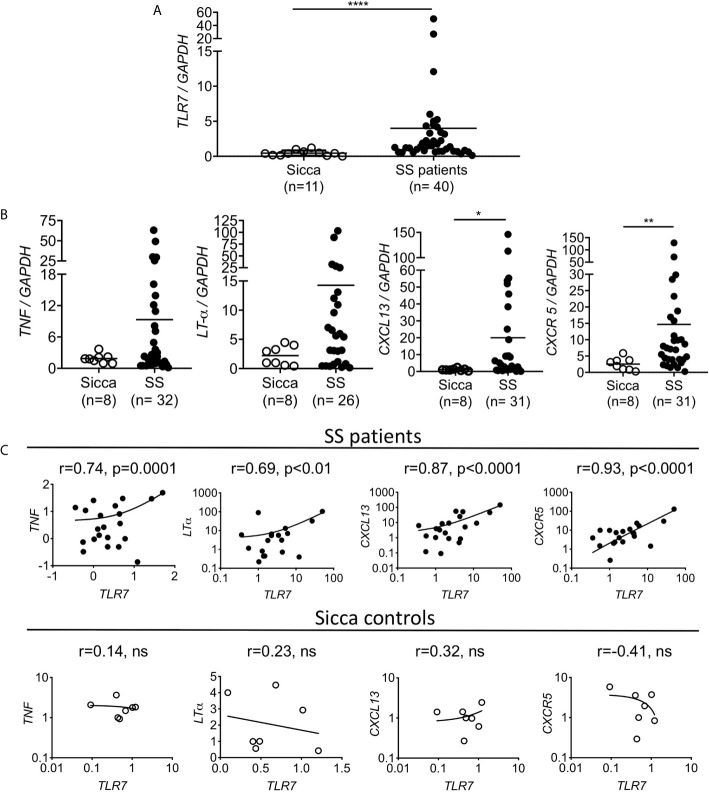
Increased TLR7 expression in the SG of the SS patients is positively correlated with *TNF*, *LT-α*, *CXCL13* and *CXCR5* expression levels. RNA was extracted from the salivary glands of female patients with primary SS and age matched sicca donors and the expression levels of **(A)** *TLR7* and **(B)** *TNF*, *LT-α*, *CXCL13* and *CXCR5* were evaluated by Q-PCR. Each point represents one individual and horizontal lines correspond to mean. Number of individuals (n) per group are denoted in parenthesis. Statistical analysis was done by non-parametric Mann-Whitney test. **(C)** Correlation analysis was performed to investigate the expression relationship between *TLR7* and *TNF*, *LT-α*, *CXCL13* and *CXCR5* expression levels, and were expressed as Pearson correlation coefficient (r). Pearson’s correlation and P values are listed at the top of each mini-plot. Each point represents one individual. For SS patients (n=19-40) and for sicca controls (n=7-11). *P < 0.05, **P < 0.01, ****P < 0.0001, ns, non statistical.

Data analyses and representations were performed with Prism 8 (GraphPad Software). Data are presented as mean ± SD unless otherwise specified. For group comparisons, statistical analysis gathering more than 2 groups were performed using Kruskal-Wallis followed by Wilcoxon rank sum tests and correction for multiple comparisons using the Benjamini-Hochberg method (**Figures 1**, **2**), or one-way ANOVA followed by Tukey test (**Figures 3**, **4** and **Supplementary Figure 1E**). For 2-group comparisons, statistical analyses were performed using Mann-Whitney U test (**Figures 5A, B**, and **Supplementary Figures 1A–D**). The Pearson correlation coefficient was applied to determine the dependence of 2 variables. Differences were considered statistically significant when *P < 0.05, **P < 0.01, ***P < 0.001, and ****P < 0.0001.

## Results

### SS Phenotype in TLR8ko Mice Is TLR7-Dependent

Since current data suggest that TLR7 might be involved in SS we examined the phenotype of TLR8ko mice, which as we reported previously, by the age of 8 months develop spontaneous SLE-like disease due to increased TLR7 signaling by DCs (10, 11). Both female and male TLR8ko mice at the age of 8- and 14-months had splenomegaly compared to WT controls (**Figure 1A** and **Supplementary Figure 1A**), while TLR7/8ko mice did not (**Figure 1A**). On the contrary, no consistent salivary gland weight to body weight variation was noted among WT and TLR8ko mice for both genders (data not shown). Since, exocrine gland inflammation is a hallmark of SS patients, we examined whether 8- and 14-months old TLR8ko female mice with clinical disease of SLE show signs of sialadenitis compared to age- and gender-matched WT and TLR7/8ko mice. We found that both 8- and 14-months old female TLR8ko mice had statistically increased submandibular gland (SMG) inflammation compared to WT or TLR7/8ko mice (**Figures 1B, C**). Moreover, we also evaluated SMG inflammation in 8-month old male TLR8ko mice, and found statistically increased inflammation compared to their WT controls (**Supplementary Figures 1B, C**). Notably, 8-months old female TLR8ko (5.8 ± 2.9) and WT (1.2 ± 1.5) mice had 2- and 8-times higher score of SMG inflammation compared to male TLR8ko (2.1 ± 1.8) and WT (0.15 ± 0.2) mice, respectively. Thus, exacerbated SG inflammation in TLR8ko mice is TLR7-dependent and is more profound in females than in males.

Next, we assessed serum levels of IgG autoantibodies against SSA, SSB and RNA in mouse sera. We found statistically increased anti-SSA, anti-SSB and anti-RNA antibodies in 8- and 14-months-old female TLR8ko compared to aged-matched WT and TLR7/8ko controls (**Figure 1D**). Fourteen-months old female TLR8ko mice showed increased levels of all three autoantibodies compared to 8-months old TLR8ko mice, however, the difference did not reach statistical significance (**Figure 1D**). Notably, the levels of anti-SSA and anti-RNA were statistically increased in female WT versus TLR7/8ko mice at the age of 8-months old and 14-months old, respectively (**Figure 1D**). Similarly, 8-months old male TLR8ko mice had statistically increased levels of anti-SSA and anti-RNA autoantibodies compared to WT mice, whereas the anti-SSB levels were also increased, but the difference did not reach statistical significance (**Supplementary Figure 1D**). To determine the possibility that direct deposition of antibodies is taking place within the SG, the presence of IgG and IgM was analysed by direct immunofluorescence. We observed increased IgG and IgM deposition in SMG of female TLR8ko versus WT mice, especially at 14-month old mice, while in TLR7/8ko mice IgG and IgM depositions were almost undetectable (**Figure 1E**). Thus, these results demonstrate that TLR8ko mice have increased serum levels of anti-SSA, anti-SSB and anti-RNA autoantibodies and deposition of IgG and IgM in the SMG compared to WT mice, and these phenomena are TLR7-dependent, since they are absent in double TLR7/8ko mice.

We noticed that the observed SS phenotype was generally more profound in female than in male mice and we wondered whether this difference could be attributed to TLR7 levels. Therefore, we evaluated TLR7 mRNA levels in the SMG of female and male WT and TLR8ko mice by quantitative PCR. *Tlr7* expression was almost 2-times higher in female than in male TLR8ko SMG (**Supplementary Figure 1E**). Based on these overall observations we decided to focus the rest of our studies in female mice.

### Lung Inflammation in TLR8ko Mice Is TLR7-Dependent

Since lung involvement is common in SS patients (31), we next investigated whether TLR8ko mice develop lung inflammation. Lungs were harvested from 8- and 14-months old female TLR8ko, TLR7/8ko and WT mice and evaluated for inflammation by H&E staining. Lungs from TLR8ko mice showed lymphocytic infiltration, predominately in the peri-bronchial regions. Both 8- and 14-months old female TLR8ko mice had statistically significant higher lung inflammation compared to WT mice (**Figures 2A–C**). However, TLR7/8ko mice did not show any signs of lung inflammation (**Figures 2A–C**). Additionally, 14-months old WT mice also showed small areas of lymphocytic infiltrates and had statistically significant higher lung inflammation compared to age-matched TLR7/8ko mice (**Figures 2B, C**). Hence, TLR8ko mice develop TLR7-dependent lung inflammation.

### Development of ELS in TLR8ko SMG

In SS patients, salivary ELS develop in 30-40% of patients (32, 33), thus we examined the presence of ELS features within the inflammatory foci, such as T/B cells segregation, as well as the presence of DCs and HEV. In female TLR8ko mice we observed T and B cell lymphocytic aggregates, in close proximity with DCs, while in WT mice these structures were predominantly absent (**Figure 3A**). No ELS were observed within the SMG of TLR7/8ko mice (data not shown). Moreover, immunofluorescent staining for HEV with MECA-79 revealed that some of the inflammatory foci in TLR8ko SMGs contained HEV that were colocalized within the areas of T and DC aggregates (**Figure 3B**). In order to further validate the increased presence of HEV in TLR8ko mice, we also evaluated the mRNA expression of the HEV markers glycosylation-dependent cell adhesion molecule-1 (*Glycam1*) and glucosaminyl (N-Acetyl) transferase 1 (*Gcnt1*) by Q-PCR in SGM derived from WT and TLR8ko mice. We noticed a statistically increased expression of both HEV markers in TLR8ko versus WT mice (**Figure 3C**). Thus, 8-months old TLR8ko mice develop ELS that contain B and T cells, DCs and HEV in their SG inflammatory foci, while these structures are generally absent in age and sex matched WT mice.

### Increased Expression of Cytokines and B Cell Markers in TLR8ko SMGs Is TLR7-Driven

To investigate if the increased SG inflammation in TLR8ko mice was accompanied by local expression of cytokines and B cell markers, RNA was extracted from the SMGs of 8- and 14-months old female WT, TLR8ko and TLR7/8 ko mice and evaluated by quantitative PCR. The expression of the inflammatory cytokines *Il-6*, *Tnf* and *Lt-α* was significantly elevated in 8- and 14-months old female TLR8ko mice compared to age and sex matched WT controls, while TLR7/8ko mice showed normal levels of all three cytokines (**Figure 4A**). We also evaluated *Ifnβ*, but the expression levels were very low to drive reliable conclusions (data not shown). In addition, since B cells contribute in the pathogenesis of SS (34), we also evaluated the expression of the B cells markers CXCL13, CXCR5 and B cell activation factor (BAFF). CXCL13 directs B-cell chemotaxis and is elevated in several autoimmune diseases, including SS (35), CXCR5 is a chemokine receptor mainly expressed on B cells and a subset of T cells, called follicular B helper T cells, and binds the chemotactic ligand CXCL13 (36), while BAFF is central to the cross talk between early activation of the immune system and the stimulation of autoreactive B cells (37). The *Cxcl13* and *Cxcr5* mRNA levels were considerably increased in the SMGs of 8- and 14-months old female TLR8ko mice versus WT or TLR7/8 controls (**Figure 4B**). Regarding *Baff*, although there were no obvious differences at 8-months old mice, there was significantly increased *Baff* expression levels in 14-months old TLR8ko SMGs compared to WT or TLR7/8ko mice (**Figure 4B**). Notably, 14-months old WT mice also showed statistically increased *Cxcl13* and *Cxcr5* expression levels compared to TLR7/8ko mice (**Figure 4B**). Thus, TLR7-signaling is the mediator for the observed increased levels of *Il-6*, *Tnf* and *Lt-α* cytokines and B cells markers *Cxcl13*, *Cxcr5* and *Baff* in TLR8ko SMG.

### Increased Cytokine and Chemokine Expression in the SG of SS Patients Is Correlated to TLR7 Expression

To gain insights into the potential association of TLR7 in the development of SS in humans, we evaluated the expression of *TLR7* in the salivary glands of 40 female patients with primary SS and 11 female age matched sicca controls using quantitative PCR. We found that *TLR7* expression was significantly higher in the salivary glands of SS patients compared to sicca controls (**Figure 5A**). We also assessed the expression of *TNF*, *LT-α*, *CXCL13* and *CXCR5*, all of which were found to be increased in the SMGs of TLR8ko mice that develop signs of SS. All four genes were higher expressed in SS patients than in sicca controls, whereas the difference was statistically significant for *CXCL13* and *CXCR5*, but not for *TNF* and *LT-α* (**Figure 5B**). Next, correlation analyses were performed to investigate the relationship between *TLR7* expression and *TNF*, *LT-α*, *CXCL13* and *CXCR5* levels. *TLR7* mRNA levels were positively correlated with *TNF* (r=0.744, p<0.0001), *LT-α* (r=0.679, p<0.0019), *CXCL13* (r=0.875, p<0.001) and *CXCR5* (r=0.923, p<0.0001) expression, while no correlations were found in sicca controls (**Figure 5C**). Hence, SS patients show increased levels of cytokines and B cell markers that are positively correlated to TLR7 expression.

## Discussion

The importance of TLR signaling is well established in many autoimmune diseases, especially the implication of TLR7 in SLE both in humans and in mice. However, the role of TLRs in SS is poorly understood. We demonstrated previously that *in vivo* TLR8 restrains TLR7 on DCs, and that TLR8ko mice on the C57BL/6 background develop spontaneous lupus due to increased TLR7 signaling by DCs, while TLR8ko B cells or macrophages retain normal TLR7 signaling (10, 11, 26). Here we show that TLR8ko mice also develop SS that is characterized by sialadenitis, lung inflammation, increased SSA and SSB autoantibody production, and within the SG deposition of IgG and IgM immune complex, increased cytokine and chemokine production and presence of ELS. Interestingly, all these phenotypes are absent in double TLR7/8ko mice indicating that the associated SS phenotype in TLR8ko mice is TLR7-dependent. In addition, by studying minor SG of female patients with primary SS we found that *TLR7*, *CXCL13* and *CXCR5* mRNA expression levels were significantly higher in diseased versus sicca controls, and that *TLR7* expression was positively correlated to *TNF*, *LT-α*, *CXCL13* and *CXCR5* expression levels. Thus, we describe a new mouse model of SS in which disease development is TLR7-driven and provide evidence that TLR7 is also implicated in the SG inflammation of SS patients. Therefore, blocking TLR7 signaling might have therapeutic utility for the progression and treatment of SS.

Previous studies have suggested an association of TLR7 with the pathophysiology of SS (38). TLR7 stimulated B cells from primary SS patients secrete increased amounts of various cytokines and show upregulated levels of surface markers (39). Type I IFN positive plasmacytoid DCs (pDCs) and monocytes isolated from peripheral blood mononuclear cells of primary SS patients show upregulation of TLR7, as well as, the downstream signaling molecules MyD88 and IRF7, and the DC maturation marker, radical S-adenosyl methionine domain containing 2 (RSAD2) (40). Moreover, monocyte-derived DCs from patients with primary SS exhibit enhanced maturation upon stimulation with the TLR7/8 agonist (CL097) compared to healthy controls (41). In accordance, our previous studies shown that both conventional DCs (cDCs) and pDCs from TLR8-deficient mice show increased expression of TLR7 that is accompanied by increased signaling upon stimulation with TLR7 agonists, while in TLR8ko macrophages or B cells TLR7 signaling is normal (26). Moreover, we demonstrated here that TLR7 signaling is pivotal for the development of associated SS in TLR8ko mice since double TLR7/8ko mice were protected, and that sialadenitis was more profound in female than in male mice. This sex bias could be associated with increased TLR7 expression in TLR8ko female versus male mice. Indeed, our present studies revealed increased TLR7 mRNA levels in the SMG of TLR8ko female versus male mice. Moreover, studies in our lab have also demonstrated increased protein levels of TLR7 in the pDCs and DCs of TLR8ko female versus TLR8ko male mice (unpublished data). Actually, a striking aspect of SS and SLE is the sexual bias, with women being affected 10 times more than men. While this female bias remains poorly understood, the X chromosome dose effect could be suspected to account for the disease. In this aspect, the TLR7 gene is located on the X chromosome and may govern gender differences in the development of systemic autoimmune diseases. In fact, recent independent studies on SS patients showed an increased prevalence of 47, XXX trisomy in women, as well as Klinefelter’s syndrome (47, XXY) in men (42, 43). Moreover, rare X chromosome abnormalities have been found among patients with either SS or SLE (44). In accordance, our present studies with female primary SS patients revealed an increased expression of *TLR7* within the minor SGs that was positively associated with *TNF*, *LT-α*, *CXCL13* and *CXCR5* expression levels. Taken together, these data demonstrate that TLR7 is likely to play an important role in the chronic inflammatory environment observed in SS disease.

ELS often develop in target organs of autoimmune diseases, including the salivary glands of patients with SS or animal models of SS (45). These structures are characterized by clusters of B and T lymphocytes, development of HEV and differentiation of follicular DC networks, while their formation and maintenance dependents on the ectopic expression of lymphotoxins and lymphoid chemokines such as CXCL13. We showed here that TLR8ko mice develop ELS within their SMG that consist of B and T cell clusters, and presence of DCs. Moreover, they show increased differentiation of HEV, consistently with enhanced expression of the HEV markers *Glycam1* and *Gcnt1* in the SMG of TLR8ko mice compared to WT or TLR7/8ko mice. Hence, TLR7 signaling is pivotal for the formation of ELS and HEV in the SMG of TLR8ko mice. In line with our findings, it has been reported that MyD88 signaling is central in the formation of HEV in the ELS of the SMG and the development of autoimmune sialadenitis in two mouse models of associated SS, the lupus prone B6/lpr and the diabetic NOD mice (46).

CXCL13 is considered as a biomarker in SS, since 74% of patients with SS display elevated CXCL13 in sera, saliva or both. Serum CXCL13 levels correlate with the extent of salivary gland inflammation and lymphoid organization (35, 47). Moreover, the CXCL13-CXCR5 axis plays a central role in regulating B cell aggregation and organization, and local expression of CXCL13 within the SG has been previously associated with the degree of cellular organization of ELS in patients with SS and animal models of the disease (47, 48). Here, we demonstrated that the formation of ELS in the SMGs of TLR8ko mice is accompanied by significantly increased levels of *Cxcl13* and *Cxcr5* within the SMG compared to WT mice. In contrast, the expression levels of *Cxcl13* and *Cxcr5* were minimal and the formation of ELS were absent within the SMG of double TLR7/8ko mice, suggesting that TLR7 signaling is vital in controlling CXCL13 expression. Moreover, we also demonstrated that in primary SS patients the increased expression of TLR7 within the minor SGs was positively associated with increased *CXCL13* and *CXCR5* expression levels. Remarkably, and in agreement with our findings CXCL13 production by human monocytes upon stimulation with ssRNA requires TLR7 activation of pDCs and secretion of type I IFNs (49).

B cells have an important role in SS, as detection of autoantibodies against SSA or SSB is one of the diagnostic criteria found in a majority of patients. Here, we found increased levels of SSA and SSB autoantibodies, as well as autoantibodies against RNA, in TLR8ko mice, while this increase was absent in double TLR7/8ko mice. Moreover, BAFF regulates B lymphocyte proliferation and survival, and has an important role in autoimmunity since BAFF transgenic mice develop lupus-like disease, and with increasing age they also develop a second pathology reminiscent to SS (50). In humans, SS also correlates with elevated levels of circulating BAFF, as well as a dramatic upregulation of BAFF expression in inflamed SGs. In accordance, in SMGs of 14-months old TLR8ko mice we found statistically increased *Baff* expression levels compared to WT mice. In contrast, TLR7/8ko mice had normal levels of *Baff* expression within their SMGs, indicating that the increased *Baff* expression in TLR8ko mice is TLR7-dependent. Noteworthy, BAFF is the target of the only currently approved biological therapy for SLE, which also provide therapeutic benefits in patients with pSS without major side effects (51).

In summary, we identified a novel role for TLR7 signaling in driving associated SS development in mice. We also provide evidence for elevated TLR7 expression within the SG of primary SS patients and a positive correlation to inflammatory markers (CXCL13, CXCR5, TNF and LT*-α*), which have been previously reported to contribute to SS disease and that were also elevated in the SMG of TLR8ko mice. Moreover, the TLR8ko mice is a new animal model for spontaneously associated SS that is TLR7-dependent, has many similarities with the observations reported in patients with SS, and as such is expected to facilitate further studies to clarify deeper the pathophysiology of SS. This work has important implications for therapeutics, since blocking the TLR7 pathway might prove to be a novel strategy for preventing and/or treating SS disease.

## Data Availability Statement

The original contributions presented in the study are included in the article/**Supplementary Material**. Further inquiries can be directed to the corresponding author.

## Ethics Statement

Ethical review and approval were not required for the study on human participants in accordance with the local legislation and institutional requirements. The patients/participants provided their written informed consent to participate in this study. The animal study was reviewed and approved by Comité National de Réflexion Ethique sur l’Expérimentation Animale.

## Author Contributions

YW, AR-Q, LC, NK, and LM conducted experiments and analyzed data. LA conceptually designed the study, analyzed the data and overall supervised the project. AN and CM designed and conducted the experiments with human samples. LA wrote the manuscript with critical appraisal from CM. All authors contributed in discussion and interpretation of experiments. All authors contributed to the article and approved the submitted version.

## Funding

This work received funding to LA from the Fondation Arthritis (Research Grant 2015-Proposal) and the Agence Nationale de la Recherche (ANR-18-CE15-0022). NK was supported by the Ministère de l’Enseignement Supérieur et de la Recherche and YW by the China Scholarship Council (CSC 201706140151). We also acknowledge institutional support from CNRS, INSERM and Aix-Marseille University.

## Conflict of Interest

The authors declare that the research was conducted in the absence of any commercial or financial relationships that could be construed as a potential conflict of interest.
