# Supplementary figures and images for: TLR7 Signaling Drives the Development of Sjögren’s Syndrome
Source: Front Immunol. 2021 May 24;12:676010. doi: 10.3389/fimmu.2021.676010 (PMC8183380; doi:10.3389/fimmu.2021.676010)

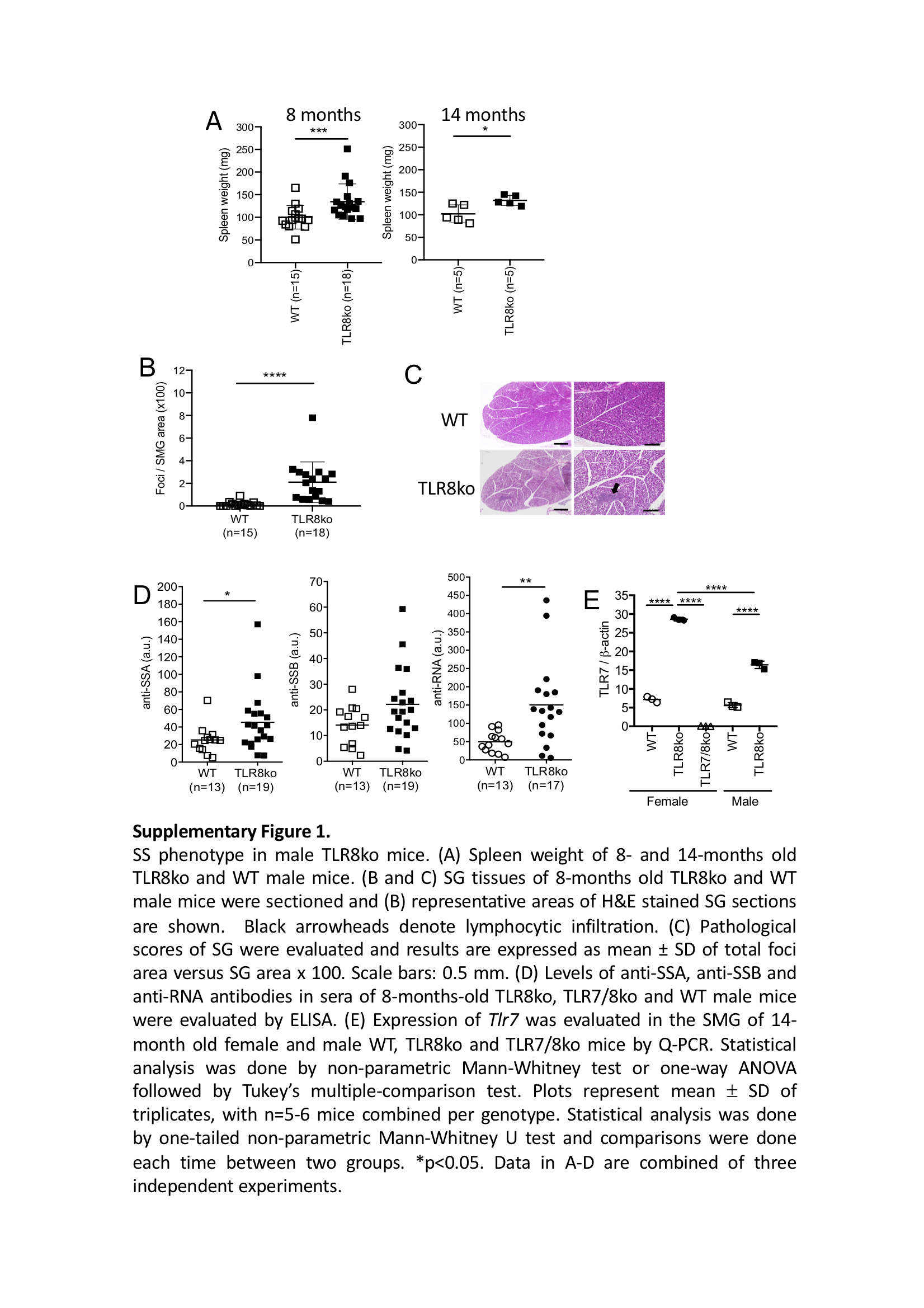

Supplement: Supplementary file 1 [file Image_1.tiff]
