# Supplementary material for: TLR7 Signaling Drives the Development of Sjögren’s Syndrome
Source: Front Immunol. 2021 May 24;12:676010. doi: 10.3389/fimmu.2021.676010 (PMC8183380; doi:10.3389/fimmu.2021.676010)
Supplement: Supplementary file 2 [file Table_1.docx]

| **Gene** | **Forward (5’→3’)** | **Reverse (5’→3’)** |
| --- | --- | --- |
| Mouse *Tlr7* | TGGCTCCCTTCTCAGGATGA | CCGTGTCCACATCGAAAACA |
| Mouse *Tnf* | TTCTATGGCCCAGACCCTCA | CAGCTGCTCCTCCACTTGGT |
| Mouse *Lt-α* | TGTTCTCTGGGAAATCGTGGA | TTTCTGCAAGTGCATCATCGTT |
| Mouse *Cxcl13* | CTCCAGGCCACGGTATTCTG | CCAGGGGGCGTAACTTGAAT |
| Mouse *Cxcr5* | TGGATGACCTGTACAAGGAACTG | AGTAAGGGTCCCTCGACTGT |
| Mouse *Baff* | TGCAAGCAGACCTGATGAAC | CCGGTGTCAGGAGTTTGACT |
| Mouse *Glycam1* | AAGACTCAGCCCACAGATGCCA | CTCTGAAGATGGAAGGCTCCTTG |
| Mouse *Gcnt1* | CCTGGAAACTGAGAAGATGCCTC | CACGAAGTAGGCACTGCCTGAA |
| Mouse *β-actin* | TGGAATCCTGTGGCATCCATGAAACC | TAAAACGCAGCTCAGTAACAGTCCG |
| Human *TLR7* | AAATGGTGTTTCCAATGTGGACA | GCCCCAAGGAGTTTGGAAAT |
| Human *TNF* | CTCTTCTGCCTGCTGCACTTTG | ATGGGCTACAGGCTTGTCACTC |
| Human *LT-α* | ACACCTTCAGCTGCCCAGACTG | TCCGTGTTTGCTCTCCAGAGCA |
| Human *CXCL13* | TATCCCTAGACGCTTCATTGATCG | CCTTCGCTTGAGGGTCCACA |
| Human *CXCR5* | TGAAGTTCCGCAGTGACCTGTC | GAGGTGGCATTCTCTGACTCAG |
| Human *GAPDH* | CAACGGATTTGGTCGTATT | GATGGCAACAATATCCACTT |

**Supplementary Table 1.** Primers used for Q-PCR.
